# Orbital metastasis as the primary manifestation of pancreatic carcinoma: a case report and literature review

**DOI:** 10.1186/s12886-022-02337-7

**Published:** 2022-03-12

**Authors:** Tatsuro Yokoyama, Aric Vaidya, Hirohiko Kakizaki, Yasuhiro Takahashi

**Affiliations:** 1 Department of Oculoplastic, Orbital & Lacrimal Surgery, Aichi Medical University Hospital, 1-1 Yazako-Karimata, Nagakute, Aichi 480-1195 Japan; 2 Department of Oculoplastic, Orbital & Lacrimal Surgery, Kirtipur Eye Hospital, Kathmandu, Nepal

**Keywords:** Pancreatic carcinoma, Metastatic orbital tumor, Diplopia, Proptosis

## Abstract

**Background:**

Orbital metastasis from pancreatic tumors is extremely rare, and its clinical characteristics are still unclear.

**Case presentation:**

Our case was a 73-year-old female who noticed diplopia on right gaze 3 months before referral to us. Imaging studies demonstrated a mass involving the lateral rectus muscle in the right orbit. The results of pathological examination of an excised specimen corresponded to poorly differentiated adenocarcinoma. Systemic work-up revealed pancreatic carcinoma with peritoneal metastasis. The patient underwent chemotherapy. We reviewed literature on similar cases and found 19 reported cases of pancreatic tumors metastasizing to the orbit. The results of our review indicate a tendency for formation of solitary mass without bony erosion, delayed detection of the primary pancreatic carcinoma, and poorer prognosis of such tumors, compared to metastatic orbital tumors from other lesions.

**Conclusions:**

We report a rare case of metastatic orbital tumor from an unknown primary pancreatic carcinoma. Clinical characteristics of cases with metastatic pancreatic tumors seem to be different from those with metastatic tumors from the other lesions. Pancreatic tumors are frequently asymptomatic in an early stage, leading to delayed detection of the primary pancreatic carcinoma and poorer prognosis.

## Background

Metastatic orbital tumors are rare entities and account for 1.5 to 12% of orbital tumors [1]. The common primary sites of these tumors are the breast, lung, prostate, and skin (melanoma) [1–6]. On the contrary, orbital metastasis from pancreatic tumors is extremely rare.

Here, we report a rare case of metastatic orbital tumor from pancreatic carcinoma without a known history of a primary lesion at the initial presentation and the result of our literature review.

## Case presentation

A 73-year-old female complained of diplopia on right gaze 3 months before referral to us. She was consulted with a neurosurgeon at another hospital, who suspected right 6^th^ cranial nerve palsy. Magnetic resonance imaging (MRI) showed a right orbital mass. The doctor followed-up her for 1 month, but the restriction of abduction deteriorated and there was development of proptosis on the right side. She did not have any history of ocular or systemic disease, or family history.

**Fig. 1:**
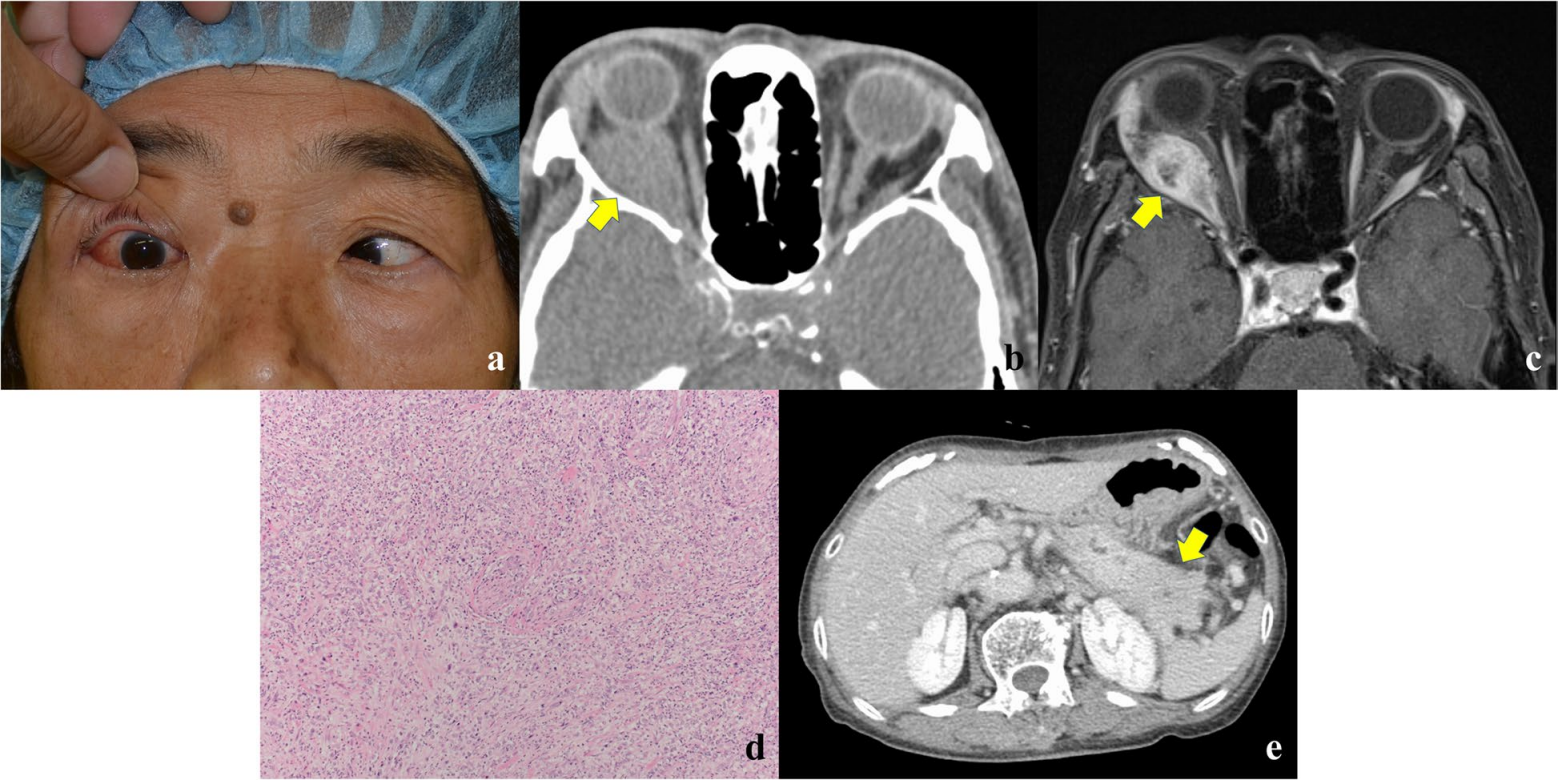
Case presentation. **a** A patient face photo showing proptosis and severe restriction of abduction in the right eye. **b** An axial computed tomographic image showing a mass involving the lateral rectus muscle without bony erosion in the right orbit (arrow). **c** An enhanced T1-weighted axial magnetic resonance image showing strong enhancement in the peripheral part of the mass (arrow). **d** Pathological examination of the specimen showing proliferation of atypical cells (hematoxylin & eosin stain; magnification, × 200). **e** An axial abdominal computed tomographic image showing enlargement of the pancreatic tail (arrow)

On the first examination, her best-corrected visual acuity was 1.0 in both eyes. Intraocular pressure was 13 mmHg in the right eye and 14 mmHg in the left eye. She did not have any field of binocular single vision in all directions of gaze, and the Hess chart showed severe restriction of adduction and abduction. There was no palpable periocular mass. The Hertel exophthalmometric examination demonstrated measurements of 20.5 mm on the right side and 15.5 mm on the left side (base, 102.5 mm) (Fig. 1a).

Computed tomographic (CT) images showed a mass in the right orbit involving the lateral rectus muscle without bony erosion (Fig. 1b). MRI revealed a mass involving the lateral rectus muscle with iso-intensity to the gray matter on T1-weighted image and heterogenous iso-to-high intensity to the gray matter on T2-weighted image. Enhanced T1-weighted MRI showed strong enhancement, especially in the peripheral part of the mass (Fig. 1c).

An excisional biopsy of the mass was performed under general anesthesia by two of the authors (YT and AV). The results of pathological examination corresponded to poorly differentiated adenocarcinoma (Fig. 1d), but the primary site was undetermined.

Systemic CT showed enlargement of the pancreatic tail with pancreatic duct dilation and small nodules in the mesenteric membrane (Fig. 1e), suspecting pancreatic carcinoma with peritoneal metastasis. The patient was consulted with a gastrointestinal physician for further examination. Blood tests demonstrated elevated carbohydrate antigen 19–9 (CA 19–9; 1141 U/mL; normal limit, < 37 U/mL), Span-1 (130 U/mL; normal limit, < 30 U/mL), carcinoembryonic antigen (CEA; 5.4 ng/mL; normal range, 0.1–5.0 ng/mL), and immunoglobulin G4 (681 mg/mL; normal limit, < 135 mg/mL). Magnetic resonance cholangiopancreatography demonstrated an enlargement of the pancreatic tail. The pancreatic duct was not depicted in the tail of pancreas but was dilated in the pancreatic body. Fine needle aspiration biopsy of the pancreatic lesion pathologically revealed the same findings to the orbital tumor.

After induction chemotherapy using FOLFIRINOX regimen at our hospital, the patient was transferred to another hospital to continue chemotherapy.

## Discussion

This report highlighted a rare case of metastatic orbital tumor from an occult pancreatic carcinoma. We reviewed literature on orbital metastasis of pancreatic tumors and found 19 reported cases (Table 1), but some of the reports did not present the details of clinical findings [7–20]. We did not include a case of uveal metastasis of pancreatic tumor in this review [21]. Pathological results included adenocarcinoma, islet cell carcinoma, and carcinoid tumor/neuroendocrine neoplasm [12–15, 17–20].

**Table 1:** Summary of the previously reported cases of metastatic pancreatic carcinoma to the orbit

Authors (Year)	Age	Sex	Side	Past history	Known primary pancreatic carcinoma (If yes, duration from diagnosis of primary carcinoma to onset)	Period from onset to first examination	Symptoms	Imaging modality	Location	Diagnostic procedure	Pathology	Other distant metastatic lesions	Treatment	Clinical course
Ferry et al. (1974) / Font et al. (1976)	-	M	-	-	-	-	-	-	-	-	-	-	-	-
Hutchison et al. (1979)	-	M	-	-	-	-	-	-	Eye or Orbit	-	-	-	-	-
		F												
Castro et al. (1982)	-	M	-	-	-	-	-	-	Eye or Orbit	-	-	-	-	-
		F												
Goldberg et al. (1990)	51	F	L	-	N	-	Eyelid edema/hyperemia, induration, motility disturbance, pain	-	-	-	-	-	-	Death 1 month after presenting with orbital metastasis
Geetha et al. (1998)	38	M	R	Nil	N	1 month	Pain, redness, diplopia, decreased vision, enophthalmos	CT	Superior orbit with incolvement of the optic nerve	FNA	Poorly differentiated adenocarcinoma	Nil	Palliative treatment	-
Chand et al. (1998)	52	M	L	Nil	N	1 month	Proptosis, decreased vision	CT	Antero-supero-medial orbit	FNA	Poorly differentiated adenocarcinoma	Liver	-	-
Gotwald et al. (2000)	53	F	R	Nil	Y (unknown)	Most recent	Headchae, swelling, proptosis, diplopia, partial visual loss	CT/MRI	Postero-supero-lateral orbit with bone erosion	Surgical resection	Islet cell carcinoma	Liver, bone	Post-chemotherapy on first examination	No recurrence at 6 months follow-up
Couch (2000)	42	F	L	Nil	Y (4 years)	a few weeks	Visual loss, diplopia, proptosis	MRI	Medial orbit involving the medial rectus muscle without bone erosion	Surgical debulking	Carcinoid tumor	Breast, liver, and mesenteric lymph nodes	Radiation and additional debulking	Death after 14 years after initial presentation
Amemiya et al. (2002)	51–68	M	R/L	-	-	-	Proptosis, limited ocular movement, palpebral tumor	-	-	-	-	Liver, stomach	-	Death 7 months after onset in one patient
		M												
Foo et al. (2010)	71	M	L	Left frontal convexity meningioma	N	6 months	Blurred vision, supraorbital ache, diplopia	CT/MRI	Postero-lateral intraconal space	Excisional biopsy	Adenocarcinoma	Nil	Radiation to the orbit	Death after 4 weeks
Pecen et al. (2012)	59	M	R	Nil	N	4 weeks	Diplopia, eyelid swelling, ptosis, proptosis	MRI	Superior orbital apex region	Exenteration	Poorly differentiated carcinoma	Liver, lymph nodes	Palliative treatment	Death 7 months after first examination
Kamieniarz (2020)	-	-	-	-	-	-	-	-	-	-	Neuroendocrine neoplasm	-	-	-
	-	-	-	-	-	-	-	-	-	-		-	-	-
	-	-	-	-	-	-	-	-	-	-		-	-	-
	-	-	-	-	-	-	-	-	-	-		-	-	-
Tsai (2021)	60	M	R	Nil	Y (1 year)	3 months	Headache, blurred vision, proptosis, ptosis, ocular pain	CT	Infero-posterior orbital space	Biopsy	Adenocarcinoma from amupulla of Vater	Multiple metastasis	High-dose 5-FU	Death 1 month later

*M* Male, *F* Female, *R* Right, *L* Left, *Y* Yes, *N* No, *CT* Computed tomography, *MRI* Magnetic resonance imaging, *FNA* Fine needle aspiration

The male to female ratio among 16 patients, including our patient with metastasis of pancreatic tumors to the orbit was 5:3, and the mean age of 11 patients was 56.2 years [7–18, 20]. These were similar to the results obtained from all the patients of orbital metastatic tumors [6, 11].

The right to left ratio among 11 patients, including our patient was almost 1:1, [11–18, 20] indicating no side-related predominance regarding orbital metastasis of pancreatic tumors.

The primary tumors are diagnosed before the onset of orbital metastasis in 85% of cases [6]. This may be due to increase in awareness and advances in medicine for early detection of malignant tumors [1, 6]. However, orbital metastasis preceded detection of the primary pancreatic carcinoma in 6 of 9 patients (66.7%) [11–15, 17, 18, 20]. This may be caused by minimal symptoms related to early stage of pancreatic carcinoma in most of the cases.

Typical radiographic findings of metastatic orbital tumors are intramuscular focal masses, bone destruction, and diffuse intraconal lesions; while a focal, solitary mass is atypical [4]. The superior and lateral quadrants of the orbit are the common regions for orbital metastasis [1]. Seven cases of metastasis of pancreatic tumors, including our case, showed a solitary mass located superiorly in 2 cases, [12, 18] medially in 1 case [15], supero-medially in 1 case [13], inferiorly in 1 case [20], supero-laterally in 1 case [14], and laterally in 1 cases [17]. Although some of the tumors involved the extraocular muscle, an intramuscular focal mass was suspected only in our case. Bone was eroded only in 1 case [14]. These results indicate that metastatic tumors of pancreatic cancer may have a tendency to show a solitary mass without bony erosion.

Symptoms and signs of orbital metastasis include proptosis/enophthalmos, diplopia, pain, vision loss, ptosis, palpable mass, subconjunctival hemorrhage, and chemosis. Proptosis and diplopia are the most common symptoms, but the manifestation of these symptoms and signs depend on the location and size of tumors [1]. Among 11 cases of metastasized pancreatic tumors, [11–18, 20] diplopia/extraocular muscle motility disturbance was the most common symptom (81.8%). Six of the 11 cases had decreased vision. Although 8 cases showed proptosis, 1 had enophthalmos. The tendency of ocular symptoms in cases of metastasized pancreatic cancer appears to be similar to that in cases of metastatic tumors from other lesions.

The prognosis in cases with orbital metastasis is generally poor [5]. The average survival for all cases is 9.3–25 months [1–3]. A previous study showed that all patients followed-up for at least 4.5 years had died at the end of the study [5]. The mean survival time tends to be longer in patients with primary breast cancer [5]. Among 7 cases of metastasized pancreatic tumors, one patient died 14 years after the initial examination [15], and another patient showed no recurrence at 6 months follow-up [14]. On the contrary, 3 patients died at 4 weeks/1 month follow-up [11, 17, 20], and the other 2 patients died 7 months after the onset or first examination [16, 18]. In addition, among 10 cases, 8 cases (80.0%) demonstrated other distant metastatic lesions [12–18, 20]. The minimal symptoms related to pancreatic carcinoma may permit insidious disease progress, resulting in poorer prognosis compared to other metastatic tumors. However, recent development of treatment modalities, such as immune checkpoint inhibitors and molecular targeted drugs may improve the survival rate.

In conclusion, we report a case of metastatic pancreatic carcinoma to the orbit and the results of literature review. Pancreatic tumors are frequently asymptomatic in an early stage, leading to delayed detection of the primary pancreatic carcinoma and poorer prognosis compared to other metastatic orbital tumors.

## Data Availability

All data are included in this paper.

## Abbreviations

CT: Computed tomography
CA 19–9: Carbohydrate antigen 19–9
CEA: Carcinoembryonic antigen
